# Explanatory machine learning for justified trust in human-AI collaboration: Experiments on file deletion recommendations

**DOI:** 10.3389/frai.2022.919534

**Published:** 2022-11-23

**Authors:** Kyra Göbel, Cornelia Niessen, Sebastian Seufert, Ute Schmid

**Affiliations:** ^1^Department of Psychology, Work and Organizational Psychology Unit, Friedrich-Alexander University of Erlangen-Nürnberg, Erlangen, Germany; ^2^Information Systems and Applied Computer Science, University of Bamberg, Bamberg, Germany

**Keywords:** distributed cognition, transactive memory, trust, forgetting, explainable AI, human-AI partnership

## Abstract

In the digital age, saving and accumulating large amounts of digital data is a common phenomenon. However, saving does not only consume energy, but may also cause information overload and prevent people from staying focused and working effectively. We present and systematically examine an explanatory AI system (Dare2Del), which supports individuals to delete irrelevant digital objects. To give recommendations for the optimization of related human-computer interactions, we vary different design features (explanations, familiarity, verifiability) within and across three experiments (*N*_1_ = 61, *N*_2_ = 33, *N*_3_= 73). Moreover, building on the concept of distributed cognition, we check possible cross-connections between external (digital) and internal (human) memory. Specifically, we examine whether deleting external files also contributes to human forgetting of the related mental representations. Multilevel modeling results show the importance of presenting explanations for the acceptance of deleting suggestions in all three experiments, but also point to the need of their verifiability to generate trust in the system. However, we did not find clear evidence that deleting computer files contributes to human forgetting of the related memories. Based on our findings, we provide basic recommendations for the design of AI systems that can help to reduce the burden on people and the digital environment, and suggest directions for future research.

## Introduction

Digital data carriers such as hard drives or cloud spaces have become important memory partners, and cognitive offloading—that is, externally saving information to reduce information processing requirements—can be used to decrease the cognitive demands of a task (Risko and Gilbert, 2016). However, in today's increasingly digitized world of work, individuals save and accumulate more digital objects in their external memory than they actually need in the short and long run. Thus, deleting or archiving irrelevant and outdated data files on a regular basis is important in several respects: It helps to reduce information overload, limits distractions, enables working in an effective, focused, and goal-oriented manner (Edmunds and Morris, 2000; Hair et al., 2007; Dabbish and Kraut, 2010; Soucek and Moser, 2010; Niessen et al., 2020b), and saves energy (Rong et al., 2016). However, often people do not delete irrelevant files, as deleting tends to be a decision under uncertainty, is effortful, and takes time. Therefore, people might benefit from an AI system designed to support individuals in deleting irrelevant digital objects in external memory (on the computer) on a regular basis. Research has shown that the transparency of system recommendations is important for willingness to use such a system and trust in a system (Pu and Chen, 2007; Wang and Benbasat, 2007; Mercado et al., 2016; Ribeiro et al., 2016; Miller, 2018; Thaler and Schmid, 2021). Thus, the AI system providing explanations plays a central role in the interaction between humans and the AI system.

To investigate whether and how an explanatory interactive AI system helps people to delete irrelevant files from their external memory (i.e., storage device), we developed an assistive AI system (Dare2Del) and conducted three experimental studies focusing on the role of explanations of Dare2Del's recommendations for users' attitudes (information uncertainty, trust), behavior (deleting files) and memory (forgetting irrelevant files). Specifically, building on the concept of distributed cognition (Hutchins, 1995; Zhang and Patel, 2006), which proposes that cognition exists both inside and outside the individual mind, prompting users to actively delete irrelevant digital objects and explaining to them why might also encourage forgetting of the related content in human memory (Sahakyan et al., 2008; Foster and Sahakyan, 2011).

Dare2Del is been developed since 2018 with the main intention to demonstrate how methods of explainable AI can be combined with interactive machine learning to keep humans in the loop in AI supported decision making (Niessen et al., 2020b; Schmid, 2021). As domain, the identification of irrelevant digital objects in the context of work has been selected for several reasons. First, in work contexts, whether a file should be deleted or not is determined by explicit laws and regulations as well as by personal preferences. Therefore, the domain is suitable for AI approaches which combine knowledge-based methods and machine learning (Muggleton et al., 2018). Second, in the context of work erroneous deletion of files might have severe consequences in contrast to private contexts and therefore, the domain is suitable to investigate the effect of explanatory and interactive AI methods on trustworthiness. Third, cloud storing of data comes with high monetary as well as environmental costs and therefore, intelligent tools to identify irrelevant files which can be deleted are of high practical relevance. Over the last years, some products which support the identification of irrelevant files have been developed, mostly in the context of file management systems, some in the context of cloud environments. For instance, Google Photos includes a feature which offers suggestions to delete photos. Suggestions are based on general characteristics such as file size, quality, unsupported format and source. In contrast, Dare2Del can take into account general rules (such as that invoices must be stored for 10 years) as well as individual preferences (such as that for presentation where a pptx exists the pdf can be deleted) which can be given as explicit rules as well as learned from feedback given to suggestions. The tool most similar to Dare2Del is offered by the teaching and learning software Canvas (https://community.canvaslms.com/t5/Canvas-Instructional-Designer/Tool-to-Identify-and-Delete-Unused-Files/ba-p/276260). However, this tool is only designed to delete unused files and empty folders directly from Canvas.

Dare2Del has been explored by five test users who work in the administration of a large company. They used a restricted version of Dare2Del on a file system which has been constructed as a mirror of their own. They used Dare2Del for a month and the general feedback has been positive. However, we are interested in a more controlled evaluation of Dare2Del in an experimental setting. For this reason, a fictitious work context had to be created which does not need specialized knowledge (e.g., accounting). At the same time, the digital objects have to be associated with some relevance such that erroneous deletion would have negative consequences. We decided to use the context of a library system where students' theses have to be archived as a suitable domain which is introduced in detail below.

Our research offers the following contributions to research on human AI collaboration: First, we provide a comprehensive analysis of both behavioral (i.e., accepting the system's suggestions) and cognitive (i.e., trust and credibility building) outcome variables. This allows us to not only identify *if* an assistive system can support users to delete irrelevant files, but also *how* it can help. Thus, our research also offers possible starting points for future improvement and individual or contextual adaptations which can help to increase deleting behavior. This is especially important, as people often do not delete irrelevant or obsolete digital objects in their working and private life. If an explainable AI system can initiate and support behavior change (i.e., lead to increased deleting of files), this might have positive consequences for individuals' stress levels and performance, but also for organizational effectiveness and energy saving. Moreover, we aim to explore underlying mechanisms of action and explain *why* explanations might be beneficial and help to change behavior (i.e., lead to increased deleting of files): We propose that explanations reduce information uncertainty, which in turn leads to more acceptance of the AI system's recommendations and the deletion of the proposed files. An understanding of these mechanisms informs design and interventions to enhance trust, credibility and behavior change in human-AI interaction.

Second, our research adds to the literature on distributed cognition by testing the assumption that an action in external memory (i.e., digital storage devices) has consequences for the corresponding internal mental representation. Previous research has already shown that external memory is used to store information outside ourselves and that this information is still connected to our memory (Sparrow et al., 2011). However, whether actively deleting information in the external memory can facilitate human forgetting of the connected memory has not been empirically investigated yet. To prove and extend existing research on the theory of distributed cognition, we ask whether deleting a digital object—especially when being convinced about why it should be deleted—also prompts forgetting of the corresponding memory content.

### Theoretical background

#### The role of explanations

An essential prerequisite for cooperative interaction between humans and AI systems is that system decisions are transparent and comprehensible (Muggleton et al., 2018). This requirement is most obvious in the context of machine learning, particularly black-box systems such as deep neural networks. Consequently, explainable AI (XAI; Miller, 2018) has been established as a new area of research, providing methods to make the decisions of machine-trained models more transparent. Several methods for highlighting the relevance of input features have been developed. For instance, visualizing the regions in an input image that had the strongest impact on the classification decision can help to identify overfitting (Lapuschkin et al., 2019). One of the most well-known methods is LIME (Ribeiro et al., 2016)—a model-agnostic method which can be applied not only to image data but also to text. XAI methods providing information about relevance are primarily helpful to model developers, and are often not informative enough for domain experts and do not provide information helpful for end-users (Schmid, 2021). For instance, in medical diagnostics, highlighting might reveal that a model that returns a specific tumor type was right for the wrong reasons because the relevant information used is some textual mark at the image border. For experts, more expressive explanations such as rules or natural language are often more helpful. For instance, the decision between two different severity classes for a tumor might depend on spatial relations such as intrusion into fat tissue or quantifications such as the number of metastases (Bruckert et al., 2020).

Explanations serve to provide reasons for an observed state of affairs (Keil, 2006; Asterhan and Schwarz, 2009; Lombrozo, 2016). Often, causal explanations also serve to justify decisions made, i.e., provide reasons why a decision is “right” (Keil, 2006; Biran and Cotton, 2017). In the field of recommender systems, different types of explanations, in particular feature-based, personalized, and non-personalized explanations, have been identified and empirically investigated in terms of their effectiveness for recommending movies (Tintarev and Masthoff, 2012). In the context of recommender systems, an extensive user study demonstrated that explanations increase willingness to use the system again and that trust in system recommendations reduces cognitive effort (Pu and Chen, 2007).

#### Distributed cognition

Distributed cognition describes the phenomenon that knowledge exists not only inside the individual, but also in his or her surroundings and within a more complex context—for example, the social, physical, or digital environment (Hutchins, 1995; Zhang and Patel, 2006). These different knowledge domains are interconnected and can influence each other not only in individual, but also in broader collective and cultural contexts (Hoskins, 2016; Sutton, 2016). Therefore, they benefit from being analyzed and treated as a holistic system. Phenomena such as saving-enhanced memory (Storm and Stone, 2015) or the photo-taking impairment effect (Henkel, 2014; Soares and Storm, 2022) show that our digital environments can be used to outsource information and provide cognitive relief (Clark and Chalmers, 1998).

Surprisingly, most basic research on human-computer interaction has not explicitly attempted to investigate conditions and outcomes of these cross-connections and information transfer processes, and the ways that people use external anchors, tools, and storage options to support and relieve their cognitive resources are rather poorly understood (Perry, 2003). We argue that analyzing the connections between internal (i.e., human memory) and external (i.e., computer memory) cognition might not only lead to a better understanding of how the different domains are coordinated and connected; it would also provide an important basis for recommendations on how human-computer interaction processes can be supported. The fact that cognitions are not only distributed, but also connected, makes it possible to determine several starting points for possible interventions. Interventions with respect to dealing with large amounts of data and information overload could start either with the user or with the computer system. For example, a reduction in load could be achieved by deleting files, which externally limits the amount of information, removes potential distractors, organizes the work environment, and therefore contributes to mental relief (Chen et al., 2012). This could further help individuals stop distracting, task-irrelevant thoughts, focus on their actual work tasks, and improve well-being (e.g., Randall et al., 2014; Kluge and Gronau, 2018; Niessen et al., 2020b; Göbel and Niessen, 2021).

In this way, the concept of distributed cognition is important from various perspectives and provides an appropriate framework for comprehensively examining human-computer interactions and developing, designing, and optimizing corresponding assistive systems.

### Development of hypotheses and research questions

As causal explanations show that there are relevant and intelligent considerations behind the system's suggestions (e.g., Keil, 2006; Biran and Cotton, 2017), we propose that explanations make it more likely that individuals will delete the proposed files (Hypothesis 1a). Moreover, we aim to replicate the finding that explanations lead to more trust in the system. Trust is defined as the willingness to rely on a technical system in an uncertain environment (Komiak and Benbasat, 2004; Meeßen et al., 2020) and has two components, one of which is more emotional and affective and one of which is more cognitive. *Affective trust* describes the user's feelings while relying on the technical system, whereas *cognitive trust* can be seen as the system's perceived trustworthiness (Komiak and Benbasat, 2006; Meeßen et al., 2020). Both affective and cognitive trust have been shown to have positive effects on intentions to adopt and work with technical agents (Meeßen et al., 2020) as well as on work outcomes and well-being (Müller et al., 2020), and thus should be considered when evaluating such systems. In line with previous research demonstrating that transparency is conducive to the development of trust (e.g., Pu and Chen, 2007; Pieters, 2011; Shin, 2021), we assume that providing explanations increases both affective and cognitive trust ratings (Hypothesis 1b).

Another important factor in this context is credibility. Described as the believability of information and its source (e.g., Fogg et al., 2001), credibility has been identified as one of the strongest predictors of trust in information systems at work (Thielsch et al., 2018). We assume that explanations generally increase the comprehensibility and transparency of the system's decisions. By providing information on *why* the system's suggestions are valid, users can better understand the reliability of the underlying processes. This should lead to increased credibility ratings (Hypothesis 1c).

Furthermore, explanations can reduce information uncertainty (Van den Bos, 2009), here the lack of information about why the system considers a file irrelevant (“why am I getting this particular file suggested”), and thus increase the likelihood of accepting the system's recommendations. As information uncertainty is often experienced negatively (e.g., Wilson et al., 2005) and can lead to ruminative thinking (Kofta and Sedek, 1999; Berenbaum et al., 2008), it might also negatively impact trust and credibility. Therefore, we not only hypothesize that explanations directly reduce information uncertainty (Hypothesis 1d), but also that information uncertainty in the system's proposals mediates the effect of explanations of the system's proposals on its acceptance (Hypothesis 2a), trust (Hypothesis 2b), and credibility (Hypothesis 2c).

It has already been shown that person-situation interactions predict how people deal with too much information in the related field of thought control (Niessen et al., 2020a). Building on these findings, we also assume that there are individual differences in the extent to which explanations support individuals' deletion of irrelevant files, trust in the AI system and finding the suggestions credible. Therefore, we investigated the moderating role of conscientiousness and need for cognition on the relation between explanations and acceptance, trust and credibility. As a personality trait, the need for cognition refers to people's tendency to engage in and enjoy thinking (Cacioppo and Petty, 1982). Individuals with a high need for cognition seek out for information to make sense of stimuli and events. Such individuals enjoy situations in which problem solving and reflection are required (Cacioppo et al., 1996). Therefore, we propose that individuals high in need for cognition have a stronger preference for thinking about the explanations, which helps them to delete irrelevant files (Hypothesis 3a), build trust (Hypothesis 3b) and credibility (Hypothesis 3c) and to reduce information uncertainty (Hypothesis 3d).

Conscientiousness is one of the Big Five personality dimensions (Barrick and Mount, 1991; Costa et al., 1991; Costa and McCrae, 1992). Conscientiousness includes the will to achieve, self-motivation, and efficaciousness, but also a dependability component that is related to orderliness, reliability, and cautiousness. We expect that conscientious individuals read and think about the explanations more deeply, as they are more cautious than less conscientious individuals. Moreover, individuals high in conscientiousness might find the explanations helpful for achieving their work goals, as deleting irrelevant files has positive consequences in terms of reduced information overload, and distractions. Therefore, we propose that conscientiousness moderates the impact of explanations on deletion of irrelevant files (Hypothesis 4a), building trust (Hypothesis 4b), and credibility (Hypothesis 4c), and on reducing information uncertainty (Hypothesis 4d).

We also hypothesize that deletion is not only an action that causes digital objects to be forgotten in external memory, but may also support intentional forgetting of associated memory content (Hypothesis 5; Bjork et al., 1998; Anderson and Hanslmayr, 2014).

Sparrow et al. (2011) showed that individuals were worse at recalling information that had been stored in external memory than information that had not been stored on the computer. This indicates that individuals need to be convinced that they will not need the information designated as irrelevant in the future in order to forget: they need to trust the system. Numerous studies on directed forgetting (Sahakyan et al., 2008; Foster and Sahakyan, 2011) and motivated forgetting (for a review, Anderson and Hanslmayr, 2014) support these assumptions. Here, we explore whether explanations can help users not only delete irrelevant information but also forget it in their memory. When they are informed why a file is irrelevant, individuals can make an informed decision, and if they accept the suggestion to delete, actually forget the file as well (research question).

## The present research

We conducted three experiments to test our hypotheses. Participants regularly interacted with the AI system Dare2Del and processed deletion suggestions in all experiments. In the first experiment, we investigated how explanations affect users' attitudes (information uncertainty, trust), behavior (deleting files), and memory (forgetting irrelevant files). In the second experiment, we further enhanced the system's transparency and verifiability by giving users the opportunity to check the correctness of the suggestions. In the third experiment, we additionally varied memory processing depth of the to-be-deleted material to further elaborate and systematize effects on memory.

To estimate the required sample size, we conducted multilevel power analyses for cross-level interaction effects (Multilevel Power Tool; Mathieu et al., 2012). We elected to use an anticipated effect size in the small-to-medium range (0.25, *p* = 0.05, Power 95%) and followed parameter recommendations from Mathieu et al. (2012) and Arend and Schäfer (2019) and in order to conservatively estimate our sample sizes. Moreover, Experiment 1 (https://aspredicted.org/3YN_FYC) and Experiment 3 (https://aspredicted.org/MQT_TYG) were preregistered on aspredicted.org. All data are publicly available on OSF (https://osf.io/dk6en/).

### Experiment 1

#### Method

##### Participants

The study was conducted with 61 undergraduates (majoring in psychology; 49 female, 12 male) from a German university. Mean age was 20.30 years (*SD* = 3.00, range 18–32). Participation was voluntary and participants received course credit as compensation.

##### Materials and procedure

The experiment was programmed with SoSciSurvey software (with additional php elements), conducted online, and lasted about an hour. During the experiment, participants were in contact with the experimenter via video chat. Before starting the experiment, demographic information (age, gender, and occupation), need for cognition, and conscientiousness were assessed with a questionnaire.

At the beginning of the experiment, participants were instructed that they would be testing a library system (see Supplementary material) at the university which digitally saves and manages dissertations, diploma, bachelor's, and master's theses (cover story). First, the participants' main task was to archive students' theses. Specifically, they had to process 36 emails from students who had sent their theses along with Supplementary information (short cover letter including author name, name of thesis, type of thesis, publication year). Participants then entered the important meta-information (title, author name, type of thesis, publication year) from all 36 emails into the digital library system and saved the email attachments automatically by pressing the respective button. After each email, they received brief feedback from the system that the entry had been saved. The emails were presented in random order. To ensure that archiving the theses always involved a comparable workload, all titles had a similar structure and consisted of two technical terms (e.g., “neuroticism and burnout”).

Second, participants were instructed to interact with an assistive system (Dare2Del) that helps to keep the digital library system tidy, without outdated or duplicate theses. The assistive system Dare2Del was described as an automatic software that detects irrelevant, identical and damaged files and suggests them for deletion. Participants were explicitly advised that the decision on whether to accept or reject the suggestion was completely up to them. Nevertheless, they were also encouraged to keep the archival system organized by using Dare2Del. While processing the emails, the assistive system popped up 12 times. Each time, a file was presented and suggested for deletion (see Supplementary material).

We systematically varied the explanation for why the file should be deleted: Six files were suggested without explanation, and for six files the assistive system provided a short explanation (three different explanations: thesis file is identical to another and was obviously saved twice; thesis file is outdated, and a newer version exists; thesis file was submitted at another university and therefore should not be in the system). Also, we systematically varied the familiarity of the files. Six of the to-be-deleted files were thesis files that the participants had previously saved into the archival system—that is, they had already entered the thesis titles into the system and saved the respective information. Six files, on the other hand, were completely new files that had not been presented before (unfamiliar files).

After processing all emails from students and suggestions for deletion from the assistive system, participants completed a recognition test. The 12 thesis file names the assistive system had suggested during the experiment (e.g., “narcissism and loneliness”) and 12 distractors (thesis file names with slightly modified titles; e.g., “egoism and loneliness”) were presented to the participants in a random order. Participants were asked to indicate whether they had processed the this exact title before or not. In this way, we assessed whether participants could correctly identify the original files they had dealt with before. At the end of the experiment, participants had the opportunity to make general comments and were then fully debriefed.

##### Research design

**Figure 1:**
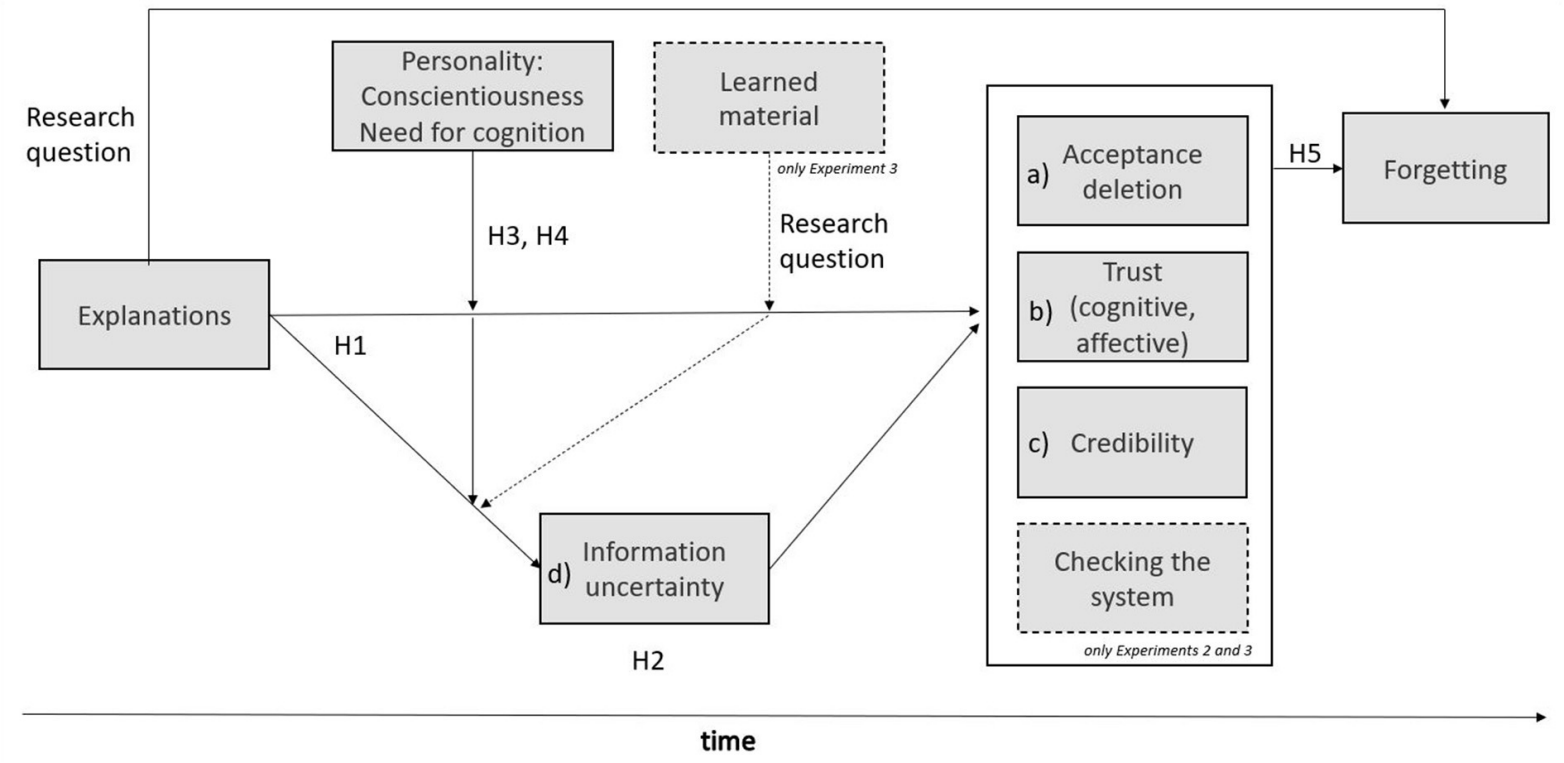
Study model.

The experimental design included an explanation condition (suggestion with explanation, coded as 1, and without explanation, coded as 0; within-person) and need for cognition and conscientiousness (between-person, see Figure 1). Need for cognition was assessed with 33 items (e.g., “I really enjoy the task of finding new solutions for problems”; Bless et al., 1994; Cronbach's Alpha = 0.92), and conscientiousness with the NEO-PI-R (60 items; Ostendorf and Angleitner, 2004; e.g., “I work goal-oriented and effectively”, Cronbach's Alpha = 0.85).

##### Dependent variables

We assessed five dependent variables. Firstly, we recorded whether the assistive system's suggestion to delete the file was accepted or not (no = 0; yes = 1). Secondly, after participants accepted or refused the suggestion, we measured trust with two components, namely cognitive trust (“I feel comfortable relying on the assistive system”) and affective trust (“I trust the assistive system completely”). The third dependent variable we measured was credibility (“The information given by the assistive system was credible”) and the fourth was information uncertainty (“I feel uncertain as to why the file was suggested for deletion, because I do not have enough information”). Trust, credibility, and information uncertainty were answered on a 5-point Likert scale ranging from 1 = *do not agree at all* to 5 = *fully agree*. Finally, we assessed the hit rate of the thesis names in the recognition test (no = 0; yes = 1).

##### Control variables

As control variable, we assessed the familiarity of the theses' titles (familiar, coded as 1, files processed by the participants; unfamiliar, coded as 0, new files not presented to participants). To consider possible effects of practice with the task, we further included a time variable in the model representing the position of Dare2Del's suggestion to delete a file (0–11). This variable makes it possible to detect systematic changes over time.

#### Results

##### Statistical analyses

Multilevel modeling and logistic multilevel modeling were used to conduct the within-person comparison of experimental conditions. Multilevel modeling presents a valuable alternative approach to traditional repeated measures analysis of variance (RM-ANOVA), as it is more robust to violations of assumptions, can handle missing data, and allows for testing more complex hierarchical structures (Cohen et al., 2003). We used R software and the packages *lme4* (Bates et al., 2015) and *mediation* (Tingley et al., 2014) to conduct our analyses. All models were two-level models, with suggestions by Dare2Del with and without explanations, familiarity of the theses' titles, deletion decisions and recognition of files in the final recognition test nested within individuals at Level 2.

The continuous Level 1 (within) predictor variable information uncertainty was centered around the person mean (Nezlek, 2012), and the continuous Level 2 (between) predictor variables (need for cognition, conscientiousness) were centered around the grand mean. Dummy-coded predictor variables were entered uncentered, as were all outcome variables for the respective models.

We applied the two-step approach to causal mediation analysis documented by Imai et al. (2010) and Tingley et al. (2014): In the first step, the mediator variable is predicted by the predictor variable, and in the second step, the outcome variable is predicted by the predictor and mediator variables. The final mediation analysis is then run using quasi-Bayesian Monte-Carlo simulations (we used 10,000 simulations each), which is superior to previous mediation approaches as it overcomes problems such as dependence on specific statistical models or restrictive assumptions (cf. Imai et al., 2010; Pearl, 2014; Tingley et al., 2014).

*N*_*Level*2_was 61, the number of the participants. Due to technical problems, we had to exclude two Level 1 datapoints, leading to an *N*_*Level*1_of 730 (61 participants × 12 deletion proposals-−2 excluded datapoints). Overall, participants accepted about one third (36%) of the system's deletion proposals. In the final recall test, 48% of the files were identified correctly. An overview of all Level 1 and Level 2 variable correlations is provided in Tables 1, 2, respectively.

**Table 1:** Experiment 1: Means, standard deviations, and correlations of Level 1 variables.

**Variable**	***M***	***SD***	**1**	**2**	**3**	**4**	**5**	**6**	**7**	**8**
1. Familiarity^a^	0.50	0.50								
2. Explanation^a^	0.50	0.50	0.00							
3. Time^b^	5.49	3.46	−0.05	0.05						
4. Deleted^a^	0.36	0.48	0.02	0.59^***^	−0.01					
5. Cognitive trust	2.31	1.03	0.12^**^	−0.02	−0.20^***^	0.16^***^				
6. Affective trust	2.38	1.09	0.13^***^	−0.02	−0.18^***^	0.17^***^	0.80^***^			
7. Credibility	2.82	1.22	0.16^***^	−0.01	−0.38^***^	0.09^*^	0.61^***^	0.64^***^		
8. Uncertainty	3.68	1.39	−0.28^***^	0.09^*^	0.26^***^	−0.09^*^	−0.56^***^	−0.61^***^	−0.67^***^	
9. Recognition^a^	0.48	0.50	0.05	−0.50^***^	0.01	−0.33^***^	0.04	0.02	−0.01	−0.02

*N*_*Level*1_ = 730.

a Dichotomous variable: “no” coded as 0, “yes” coded as 1.

b Position of deleting proposals (0–11).

* *p* < 0.05,

** *p* < 0.01,

*** *p* < 0.001.

**Table 2:** Experiment 1: Means, standard deviations, and correlations of Level 2 and aggregated Level 1 variables.

**Variable**	***M***	***SD***	**1**	**2**	**3**	**4**	**5**	**6**	**7**	**8**	**9**
1. Age	20.49	3.00									
2. Gender^a^	0.20	0.40	−0.07								
3. Conscientiousness	3.72	0.34	0.03	−0.00							
4. Need for cognition	3.39	0.52	0.13	0.21	0.20						
5. Deleted^b^^,^ ^c^	0.36	0.18	−0.06	−0.06	0.11	−0.21					
6. Cognitive trust^c^	2.30	0.67	−0.01	−0.01	0.28^*^	−0.22	0.65^***^				
7. Affective trust^c^	2.37	0.65	−0.04	−0.02	0.22	−0.23	0.74^***^	0.91^***^			
8. Credibility^c^	2.82	0.51	−0.01	−0.04	0.12	−0.24	0.51^***^	0.53^***^	0.61^***^		
9. Uncertainty^c^	3.69	0.53	0.22	−0.05	−0.10	0.24	−0.69^***^	−0.67^***^	−0.71^***^	−0.54^***^	
10. Recognition^b^^,^ ^c^	0.48	0.16	−0.04	−0.06	−0.08	0.19	0.10	0.09	0.10	−0.03	0.09

*N*_*Level*2_ = 61.

a Female coded as 0, male coded as 1.

b Dichotomous variable: “no” coded as 0, “yes” coded as 1.

c Level 1 variable aggregated on the person-level.

* *p* < 0.05,

*** *p* < 0.001.

##### Hypothesis testing

First, we tested the hypothesis that explanations lead to higher acceptance of the assistive system's suggestions, to more cognitive and affective trust, more credibility, and less information uncertainty (Hypotheses 1a–d). To do so, we calculated (logistic) multilevel regression analyses. In line with our expectations, the presence of explanations led to higher acceptance of the deletion suggestions (γ = 3.96, *SE* = 0.31, *z* = 12.63, *p* < 0.001). However, explanations did not increase trust (cognitive trust: γ = −0.02, *SE* = 0.06, *t* = −0.37, *p* = 0.709; affective trust: γ = −0.02, *SE* = 0.07, *t* = −0.24, *p* = 0.814) or the credibility of the system (γ = 0.02, *SE* = 0.08, *t* = 0.20, *p* = 0.840). Contrary to our expectations, explanations for the suggestions increased rather than decreased information uncertainty (γ = 0.21, *SE* = 0.09, *t* = 2.30, *p* = 0.021). However, it should be noted that due to simultaneous testing of up to five dependant variables, the *p*-value needs to be adjusted down to 0.01 (0.05/5; Haynes, 2013).

The familiarity of the files, which we added as a control variable to our analyses to examine possible effects of different levels of cognitive processing, led to more cognitive and affective trust, more credibility and less uncertainty (see Table 3). However, it did not affect acceptance of the suggestions. The results of the time variable revealed a decrease in cognitive and affective trust and an increase in information uncertainty over time (see also Table 3). These findings are somewhat unexpected and need to be further examined and discussed.

**Table 3:** Experiment 1: Effects of explanations on acceptance of deleting proposal, cognitive trust, affective trust, credibility, and uncertainty.

	**Acceptance of deleting proposal^a^**			**Cognitive trust^b^**			**Affective trust^b^**			**Credibility^b^**			**Uncertainty^b^**		
**Predictor**	***Est*.**	***SE***	***z***	***Est*.**	***SE***	***t***	***Est*.**	***SE***	***t***	***Est*.**	***SE***	***t***	***Est*.**	***SE***	***t***
Constant	−3.02	0.36	−8.37^***^	2.53	0.11	23.87^***^	2.55	0.11	23.50^***^	3.37	0.11	31.99^***^	3.42	0.12	28.90^***^
Time	−0.05	0.03	−1.63	−0.06	0.01	−7.00^***^	−0.06	0.01	−5.88^***^	−0.13	0.01	−11.69^***^	0.10	0.01	7.31^***^
Familiarity	0.20	0.22	0.90	0.22	0.06	3.73^***^	0.27	0.07	4.08^***^	0.34	0.08	4.37^***^	−0.74	0.09	−8.12^***^
Explanation	3.96	0.31	12.63^***^	−0.02	0.06	−0.37	−0.02	0.07	−0.24	0.02	0.08	0.20	0.21	0.09	2.30^*^

*N*_*Level*1_ = 730, *N*_*Level*2_ = 61.

a Logistic multilevel regression analysis.

b Continuous multilevel regression analysis.

*** *p* < 0.001,

* *p* < 0.05.

Second, we tested the mediating role of information uncertainty with regard to acceptance of the system's suggestions, cognitive trust, affective trust, and credibility. Information uncertainty did not mediate the effect of explanations on acceptance of the deletion suggestions (indirect effect = −0.00, 95% CI [−0.01; 0.00], *p* = 0.310). However, we found indirect effects for cognitive trust (indirect effect = −0.07, 95% CI [−0.13; −0.01], *p* = 0.020), affective trust (indirect effect = −0.09, 95% CI [−0.16; −0.01], *p* = 0.020), and credibility (indirect effect = −0.12, 95% CI [−0.22; −0.02], *p* = 0.020), but as with the results for Hypothesis 1, the direction of effects was unexpected: Explanations created *more* information uncertainty, which resulted in *less* cognitive trust, *less* affective trust, and *less* credibility. Thus, Hypotheses 2a–c were not confirmed, although they highlighted the mediating role of information uncertainty.

In the next step, we tested whether need for cognition (Hypotheses 3a–d) and conscientiousness (Hypotheses 4a–d) moderated the effect of explanations on acceptance, cognitive trust, affective trust, credibility, and information uncertainty. To do so, we calculated cross-level interactions. However, none of them turned out to be significant for either need for cognition (all *z*s/*t*s < |1.14|, all *p*s > 0.252) or conscientiousness (all *z*s/*t*s < |1.28|, all *p*s > 0.202) as a moderator. Therefore, we had to completely reject Hypothesis 3 and Hypothesis 4. We further investigated whether deleting a file led to subsequent forgetting. Confirming Hypothesis 5, deleting a file was associated with a lower recognition probability (γ = −1.69, *SE* = 0.19, *z* = −8.69, *p* < 0.001).

To explore possible effects of explanations on the subsequent accessibility of the corresponding memory traces (research question), we calculated additional multilevel regression analyses. The results revealed that the presence of explanations for a suggestion to delete a thesis file was associated with a lower probability of recognizing its title in the recognition test (γ = −2.17, *SE* = 0.22, *z* = −9.68, *p* < 0.001), thus indicating difficulties in retrieval (which corresponds to forgetting).

##### Additional analyses

As explanations were positively associated with deleting a file (Hypothesis 1a), we further conducted a two-step causal mediation analysis to test whether the act of deletion mediates the effect of explanations on subsequent forgetting. The indirect effect was not significant (indirect effect = −0.04, 95% CI [−0.09; 0.01], *p* = 0.110), but the direct effect from explanations on the recognition rate was again confirmed (direct effect = −0.46, 95% CI [−0.54; −0.37], *p* < 0.001). Unexpectedly, time and familiarity of files did not affect final recall rates.

In sum, as expected, explanations led to higher acceptance of the deletion suggestions and to more forgetting of the files. Contrary to our hypotheses, explanations did not increase trust or credibility of the system, but increased information uncertainty, which led to less trust. Moreover, over the course of the experiment, trust actually decreased and information uncertainty increased.

### Experiment 2

To further explore the surprising effects of explanations on trust, credibility, and information uncertainty, we conducted a second experiment with two major changes. First, we assumed that the negative effects of explanations on trust, credibility, and information security in Experiment 1 were because participants were not able to check the explanations and suggestions in the file system. As a result, they simply accepted the suggestions blindly, but did not trust them, did not find the system credible, and felt more uncertain. In Experiment 2, we provided the possibility to check the explanations and suggestions by looking at the files in the folder (see Supplementary material). Therefore, we were able to assess participants' trust in a more objective manner (with less checking of explanations indicating more trust). Second, we modified the kind of explanations, so that acceptance of the suggestion would imply definite and final removal (in contrast to the deletion of duplicates, where one of the original files continues to exist in the file system). Therefore, we partly changed the explanations' content (e.g., thesis was submitted at a foreign university that was not part of the literature network system).

#### Method

##### Participants

Participants were 33 undergraduates (participating in return for course credit) at a German university (27 female, five male, one non-binary). Mean age was 22.55 years (*SD* = 4.98, range 18–40).

##### Materials and procedure

The materials and procedure for the experiment were similar to Experiment 1, with two exceptions. First, participants had access to the underlying file system. They could scroll through the file list, which consisted of 50 alphabetically sorted thesis data files, and could check whether the explanations provided by the assistive system were appropriate (e.g., file was duplicate in the system). The explanations were always correct and consistent with the file system. Second, we varied the kind of explanations. One explanation stated that the file had accidently been saved twice, and that because of this, one copy had to be removed. The other explanation stated that the file was erroneously in the file system as it was submitted at a foreign university that was not part of the literature network system, and therefore suggested final removal.

##### Research design

The design was the same as for Experiment 1.

##### Dependent variables

In addition to the dependent variables in Experiment 1, we were able to assess an additional measure of trust, namely, whether participants checked the validity of the suggestions. We measured whether participants had opened the underlying file system (no = 0; yes = 1) and how much time the participants spent scrolling through and checking it (in milliseconds).

##### Control variables

As in Experiment 1, familiarity and time were included as control variables.

#### Results

##### Statistical analyses

We followed the same analytic strategy as Experiment 1. *N*_*Level*2_was 33, the number of the participants. *N*_*Level*1_was 396 (33 participants × 12 deletion proposals). An overview of the correlations of all Level 1 and Level 2 variables is provided in Tables 4, 5, respectively. Overall, participants checked the file system in 70% of all cases, and accepted about two thirds (70%) of the system's deletion proposals. In the final recall test, 43% of the files were identified correctly.

**Table 4:** Experiment 2: Means, standard deviations, and correlations of Level 1 variables.

**Variable**	***M***	***SD***	**1**	**2**	**3**	**4**	**5**	**6**	**7**	**8**	**9**	**10**
1. Familiarity^a^	0.50	0.50										
2. Explanation^a^	0.50	0.50	0.00									
3. Time^b^	5.50	3.46	−0.05	0.05								
4. Checked^a^	0.70	0.46	0.03	−0.02	−0.07							
5. Checked (time)^c^	6.67	4.40	0.03	−0.01	−0.10^*^	1.00^***^						
6. Deleted^a^	0.70	0.46	−0.02	0.09	−0.04	0.17^**^	0.16^**^					
7. Cognitive trust	2.94	1.32	0.01	0.06	0.07	0.02	−0.00	0.52^***^				
8. Affective trust	3.04	1.32	0.00	0.07	0.08	0.06	0.04	0.49^***^	0.87^***^			
9. Credibility	3.66	1.31	0.03	0.11^*^	−0.03	0.22^***^	0.20^***^	0.66^***^	0.67^***^	0.72^***^		
10. Uncertainty	2.52	1.53	−0.01	−0.13^**^	−0.05	−0.28^***^	−0.26^***^	−0.60^***^	−0.52^***^	−0.52^***^	−0.61^***^	
11. Recognition^a^	0.43	0.50	0.05	0.11^*^	0.36^***^	0.04	0.03	−0.05	−0.04	−0.05	−0.06	0.01

*N*_*Level*1_ = 396.

a Dichotomous variable: “no” coded as 0, “yes” coded as 1.

b Position of deleting proposals (0–11).

c Logarithmized, in milliseconds.

* *p* < 0.05,

** *p* < 0.01,

*** *p* < 0.001.

**Table 5:** Experiment 2: Means, standard deviations, and correlations of Level 2 and aggregated Level 1 variables.

**Variable**	***M***	***SD***	**1**	**2**	**3**	**4**	**5**	**6**	**7**	**8**	**9**	**10**	**11**
1. Age	22.55	4.98											
2. Gender^a^	0.16	0.37	−0.09										
3. Conscientiousness	3.62	0.35	−0.14	0.20									
4. Need for cognition	3.34	0.45	−0.15	0.01	0.47^**^								
5. Checked^b^^,^ ^c^	0.70	0.40	−0.07	0.19	0.30	0.32							
6. Checked (time)^c^^,^ ^d^	6.67	3.83	−0.07	0.18	0.29	0.31	1.00^***^						
7. Deleted^b^^,^ ^c^	0.70	0.24	−0.02	−0.01	−0.03	−0.05	0.36^*^	0.36^*^					
8. Cognitive trust^c^	2.94	1.00	−0.14	0.16	−0.00	−0.10	0.10	0.08	0.51^**^				
9. Affective trust^c^	3.04	1.04	−0.17	0.28	−0.05	−0.14	0.11	0.09	0.46^**^	0.94^***^			
10. Credibility^c^	3.66	0.88	−0.14	0.29	0.08	−0.03	0.36^*^	0.35^*^	0.75^***^	0.67^***^	0.74^***^		
11. Uncertainty^c^	2.52	1.02	−0.06	−0.13	−0.03	−0.25	−0.44^*^	−0.43^*^	−0.69^***^	−0.52^**^	−0.50^**^	−0.68^***^	
12. Recognition^b^^,^ ^c^	0.43	0.18	−0.32	−0.15	0.26	0.24	0.21	0.22	−0.06	−0.20	−0.32	−0.23	0.22

*N*_*Level*2_ = 33.

a Female coded as 0, male coded as 1.

b Dichotomous variable: “no” coded as 0, “yes” coded as 1.

c Level 1 variable aggregated on the person-level.

d Logarithmized, in milliseconds.

* *p* < 0.05,

** *p* < 0.01,

*** *p* < 0.001.

##### Hypothesis testing

To test Hypotheses 1a–d, we again analyzed the effect of explanations on the acceptance of deletion proposals, cognitive trust, affective trust, checking the file system, credibility, and information uncertainty. Results showed that when explanations were given, participants were more likely to delete the suggested file (γ = 0.53, *SE* = 0.25, *z* = 2.16, *p* < 0.05), considered the system more credible (γ = 0.29, *SE* = 0.10, *t* = 2.89, *p* < 0.01), and reported less uncertainty (γ = −0.40, *SE* = 0.12, *t* = −3.33, *p* < 0.001). They did not trust the system more either cognitively or affectively and there were no effects on frequency of or time spent checking the file system. However, further analyses indicated an increase in affective trust as well as less and shorter periods of checking the file system over time (see Tables 6A,B). Thus, Hypothesis 1 could only be partly confirmed. Familiarity of files did not exhibit any effects.

**Table 6A:** Experiment 2: Effects of explanations on acceptance of deleting proposal, cognitive trust, affective trust, credibility, and uncertainty.

	**Acceptance of deleting proposal^a^**			**Cognitive trust^b^**			**Affective trust^b^**			**Credibility^b^**			**Uncertainty^b^**		
**Predictor**	***Est*.**	***SE***	***z***	***Est*.**	***SE***	***t***	***Est*.**	***SE***	***t***	***Est*.**	***SE***	***t***	***Est*.**	***SE***	***t***
Constant	1.16	0.38	3.07^**^	2.71	0.20	13.58^***^	2.79	0.20	13.78^***^	3.54	0.19	18.87^***^	2.86	0.22	13.05^***^
Time	−0.04	0.04	−1.02	0.02	0.01	1.87	0.03	0.01	2.27^*^	−0.01	0.01	−0.87	−0.02	0.02	−1.24
Familiarity	−0.11	0.25	−0.44	0.04	0.09	0.42	0.01	0.09	0.17	0.08	0.10	0.81	−0.05	0.12	−0.40
Explanation	0.53	0.25	2.16^*^	0.14	0.09	1.57	0.17	0.09	1.92	0.29	0.10	2.89^**^	−0.40	0.12	−3.33^***^

*N*_*Level*1_ = 396, *N*_*Level*2_ = 33.

a Logistic multilevel regression analysis.

b Continuous multilevel regression analysis.

*** *p* < 0.001,

** *p* < 0.01,

* *p* < 0.05.

**Table 6B:** Experiment 2: Effects of explanations on checking the system.

	**Checking the file system^a^**			**Time spent checking the file system^b^^,^ ^c^**		
**Predictor**	***Est*.**	***SE***	***z***	***Est*.**	***SE***	***t***
Constant	4.27	2.07	2.06^*^	7.28	0.71	10.24^***^
Time	−0.16	0.06	−2.44^*^	−0.13	0.03	−3.79^***^
Familiarity	0.38	0.43	0.90	0.23	0.23	0.97
Explanation	−0.22	0.42	−0.51	−0.04	0.23	−0.17

*N*_*Level*1_ = 396, *N*_*Level*2_ = 33.

a Logistic multilevel regression analysis.

b Continuous multilevel regression analysis.

c Logarithmic (originally in milliseconds).

*** *p* < 0.001,

* *p* < 0.05.

In the next step, we again tested for the possible mediating role of information uncertainty (Hypotheses 2a–c). Significant mediation processes could be identified for all dependent variables: Explanations generally reduced information uncertainty, and reduced uncertainty in turn led to increased acceptance of deletion proposals (indirect effect = 0.05, 95% CI [0.02; 0.08], *p* < 0.001), more cognitive trust (indirect effect = 0.16, 95% CI [0.06; 0.25], *p* < 0.001), affective trust (indirect effect = 0.16, 95% CI [0.07; 0.26], *p* < 0.001), and credibility (indirect effect = 0.18, 95% CI [0.07; 0.30], *p* < 0.001). No indirect effect of information uncertainty was found for either opening the file system (indirect effect = 0.01, 95% CI [−0.00; 0.02], *p* = 0.150) or time spent checking the file system (indirect effect = 0.05, 95% CI [−0.02; 0.15], *p* = 0.180).

Concerning possible moderating effects of need for cognition (Hypotheses 3a–d) on acceptance of the proposals, cognitive and affective trust, checking the file system, credibility and information uncertainty, we found no significant interactions between presence of explanations and need for cognition on credibility, all *z*s/*t*s < |1.47|, all *p*s > 0.142.

**Figure 2:**
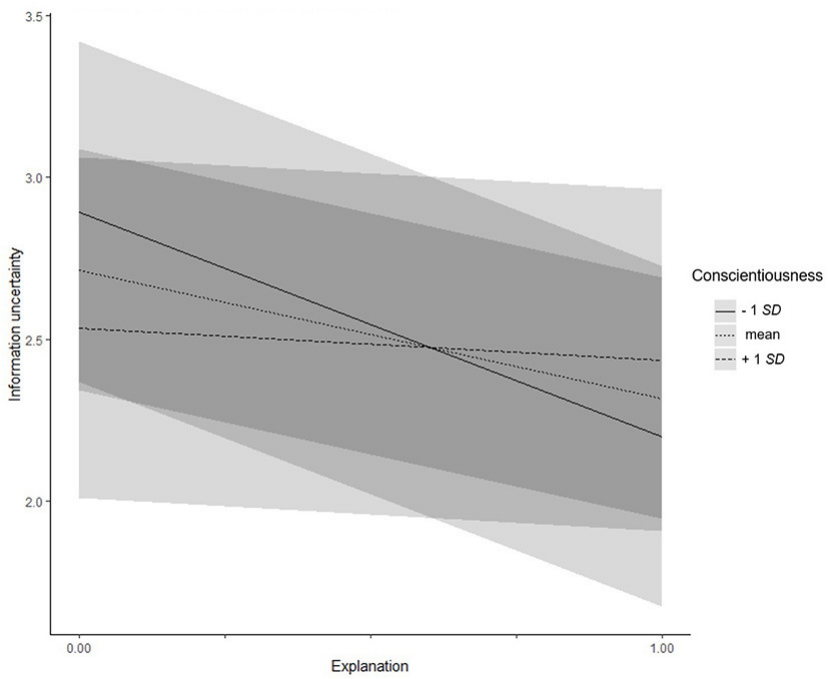
Experiment 2: Interaction effect between conscientiousness and explanations on information uncertainty. Low and high levels of information uncertainty represent one standard deviation below and above the mean, respectively.

For conscientiousness (Hypotheses 4a–d), a significant interaction effect with presence of explanations on information uncertainty was found (γ = 0.87, *SE* = 0.35, *t* = 2.52, *p* = 0.012; see Figure 2): People with lower (−1 *SD*) conscientiousness reported less information uncertainty when an explanation was provided (simple slope = −0.70, *t* = −4.15, *p* < 0.001). For people with higher (+1 *SD*) conscientiousness, no difference was found (simple slope = −0.09, *t* = −0.54, *p* = 0.591). For all other dependent variables, no effects were detected, all *z*s/*t*s < |1.88|, all *p*s > 0.061. Therefore, Hypothesis 4 could only be confirmed for information uncertainty.

Lastly, we tested whether explanations and the deletion of files led to more subsequent difficulties in recognizing the thesis titles (H5). Contrary to the results of Experiment 1, we found that explanations were associated with a higher likelihood of subsequent recognition (γ = 0.46, *SE* = 0.23, *z* = 2.00, *p* = 0.046), and deleting a file was not related to recognition at all (γ = −0.24, *SE* = 0.27, *z* = −0.92, *p* = 0.358). Therefore, Hypothesis 5 was not supported.

##### Additional analyses

We tested for differences between the two kinds of explanations. The results revealed that participants accepted more suggestions to delete duplicates (γ = 2.43, *SE* = 0.46, *z* = 5.26, *p* < 0.001) and fewer suggestions to delete files that were erroneously in the system (γ = −0.64, *SE* = 0.31, *z* = −2.08, *p* < 0.05) compared to files with no explanations. There was no effect of explanations on checking the file system. However, files with the duplicate explanation were more likely to be recognized in the recall test than files with no explanation (γ = 0.68, *SE* = 0.31, *z* = 2.23, *p* < 0.05), whereas there was no difference between files identified as erroneously in the system and the no explanation condition.

In sum, in this experiment, the possibility of checking why the system suggested a thesis for deletion led not only to more suggestions being accepted, but also to more trust over time, credibility and information uncertainty. Explanations reduced information uncertainty, which was in turn related to more trust and credibility. However, in contrast to Experiment 1, explanations also improved recall of the titles suggested for deletion, and did not promote forgetting.

### Experiment 3

The aim of Experiment 3 was to replicate the results of Experiment 2 and investigate whether explanations promote forgetting of well-known information (i.e., thesis titles). Based on research on intentional forgetting, we assumed that an explanation for why a file can be deleted should indicate to participants that the memory content connected to this file can be intentionally forgotten (“I don't need it anymore, so I can forget it”). In the previous experiments, however, we did not control for whether our participants had actually remembered the thesis titles they entered into the database. Therefore, in this experiment, one group of participants had to learn and remember the thesis titles.

#### Method

##### Participants

Experiment 3 was conducted with 73 undergraduates (55 female, 18 male) at a German university. Mean age was 23.14 years (*SD* = 3.87, range 18–36). Participation was voluntary and participants received either course credit or a financial reward (€ 15) as compensation.

##### Materials and procedure

The experimental task was similar to that in Experiment 2 but consisted of two parts. In the learning phase, participants (*n* = 32) in the learning condition were instructed to learn the six familiar file names. To ensure that the file names had been learned sufficiently, a recognition test with the file names as well as six distractor names was conducted. Only if there were no recognition errors did the main part of the experiment—archiving theses—start; otherwise, participants had to relearn the thesis titles and were then given a second recognition test. In the control condition, participants (*n* = 41) did not learn the file names before starting the main part of the experiment. The materials and procedure for the main part of the experiment were the same as in Experiment 2. However, for reasons of consistency, we used two explanations from Experiment 1, neither of which implied final and definitive removal in case of deletion (thesis file is identical to another one and was clearly saved twice; thesis file is outdated and a newer version exists).

##### Research design

The experimental design included an explanation condition (with explanation, coded as 1; without explanation, coded as 0; within-person), a learning condition (learning coded as 1, and no learning coded as 0; between-person) and need for cognition and conscientiousness (between-person, see Figure 1).

##### Dependent variables

Dependent variables and measures were the same as in Experiment 2.

##### Control variables

As in Experiment 2, familiarity and time were included as control variables.

#### Results

##### Statistical analyses

We followed the same analytic strategy as Experiments 1 and 2. *N*_*Level*2_was 73, the number of the participants. *N*_*Level*1_was 876 (73 participants × 12 deletion proposals). An overview of the correlations among all Level 1 and Level 2 variables is provided in Tables 7, 8, respectively. Overall, participants checked the file system in 71% of all cases, and accepted about two-thirds (68%) of the system's deletion suggestions. In the recognition test, 49% of the files were identified correctly. In the learning group, 76% of the files were checked, 70% were deleted, and the overall recognition rate was 54%. In the control group, participants checked 68% of the files, deleted 66%, and identified 44% of the files correctly on the final recognition test.

**Table 7:** Experiment 3: Means, standard deviations, and correlations of Level 1 variables.

**Variable**	***M***	***SD***	**1**	**2**	**3**	**4**	**5**	**6**	**7**	**8**	**9**	**10**
1. Familiarity^a^	0.50	0.50										
2. Explanation^a^	0.50	0.50	0.00									
3. Time^b^	5.50	3.45	−0.05	0.05								
4. Checked^a^	0.71	0.45	0.04	−0.07^*^	−0.05							
5. Checked (time)^c^	6.59	4.22	0.04	−0.07^*^	−0.09^**^	0.99^***^						
6. Deleted^a^	0.68	0.47	−0.01	0.18^***^	0.15^***^	0.14^***^	0.13^***^					
7. Cognitive trust	2.75	1.21	−0.03	0.11^**^	0.21^***^	−0.04	−0.06	0.52^***^				
8. Affective trust	2.84	1.22	0.02	0.12^***^	0.20^***^	0.02	−0.01	0.52^***^	0.84^***^			
9. Credibility	3.60	1.12	0.01	0.20^***^	0.16^***^	0.13^***^	0.11^**^	0.49^***^	0.52^***^	0.55^***^		
10. Uncertainty	2.87	1.44	−0.06	−0.21^***^	−0.22^***^	−0.07^*^	−0.05	−0.55^***^	−0.54^***^	−0.56^***^	−0.44^***^	
11. Recognition^a^	0.49	0.50	0.06	0.09^**^	0.40^***^	0.06	0.05	0.06	0.03	0.04	0.07	−0.07^*^

*N*_*Level*1_ = 876.

a Dichotomous variable: “no” coded as 0, “yes” coded as 1.

b Position of deleting proposals (0-11).

c Logarithmized, in milliseconds.

* *p* < 0.05,

** *p* < 0.01,

*** *p* < 0.001.

**Table 8:** Experiment 3: Means, standard deviations, and correlations of Level 2 and aggregated Level 1 variables.

**Variable**	***M***	***SD***	**1**	**2**	**3**	**4**	**5**	**6**	**7**	**8**	**9**	**10**	**11**
1. Age	23.14	3.87											
2. Gender^a^	0.25	0.43	0.30^**^										
3. Conscientiousness	3.71	0.39	0.10	0.03									
4. Need for cognition	3.42	0.40	0.09	0.31^**^	0.11								
5. Checked^b^^,^ ^c^	0.71	0.36	0.14	0.20	−0.24^*^	−0.09							
6. Checked (time)^c,d^	6.59	3.29	0.15	0.21	−0.24^*^	−0.09	1.00^***^						
7. Deleted^b^^,^ ^c^	0.68	0.27	−0.03	−0.01	−0.13	−0.19	0.31^**^	0.32^**^					
8. Cognitive trust^c^	2.75	0.87	0.08	−0.16	−0.12	−0.03	−0.00	−0.01	0.54^***^				
9. Affective trust^c^	2.84	0.89	0.11	−0.12	−0.15	−0.01	0.08	0.07	0.57^***^	0.91^***^			
10. Credibility^c^	3.60	0.74	0.10	0.10	0.04	−0.01	0.32^**^	0.32^**^	0.55^***^	0.45^***^	0.48^***^		
11. Uncertainty^c^	2.87	0.84	0.05	0.12	0.02	0.09	−0.20	−0.19	−0.63^***^	−0.49^***^	−0.49^***^	−0.39^**^	
12. Recognition^b^^,^ ^c^	0.49	0.19	−0.12	−0.07	−0.20	−0.18	0.29^*^	0.31^**^	0.03	−0.15	−0.07	−0.09	−0.03

*N*_*Level*2_ = 73.

a Female coded as 0, male coded as 1.

b Dichotomous variable: “no” coded as 0, “yes” coded as 1.

c Level 1 variable aggregated on the person-level.

d Logarithmized, in milliseconds.

* *p* < 0.05,

** *p* < 0.01,

*** *p* < 0.001.

##### Hypothesis testing

Hypotheses 1a–d proposed that explanations would lead to higher acceptance of the deletion proposals, more trust (cognitive trust, affective trust, and less verification of the suggestions), greater credibility, and less information uncertainty. The results revealed that when explanations were given, participants were more likely to delete a file (γ = 1.13, *SE* = 0.18, *z* = 6.23, *p* < 0.001), trusted the system more cognitively (γ = 0.23, *SE* = 0.06, *t* = 4.10, *p* < 0.001) as well as affectively (γ = 0.27, *SE* = 0.06, *t* = 4.88, *p* < 0.001), checked the file system less frequently (γ = −0.79, *SE* = 0.24, *z* = −3.26, *p* = 0.001), spent less time checking the system (γ = −0.54, *SE* = 0.19, *t* = −2.92, *p* = 0.004), considered the system more credible (γ = 0.43, *SE* = 0.06, *t* = 7.58, *p* < 0.001), and reported less information uncertainty (γ = −0.58, *SE* = 0.07, *t* = −7.50, *p* < 0.001). Thus, Hypothesis 1 was supported. The results did not differ between participants who had learned the file names prior to the experiment and those who had not. Also, the familiarity of file names, that is, the names of files participants had archived themselves (learning group: learned and archived) vs. unfamiliar files, i.e., files that already existed before participants started working with the literature management system, had no effect on the dependent variables (see Tables 9A,B) with one exception: Participants reported less information uncertainty when the to-be-deleted files were familiar.

**Table 9A:** Experiment 3: Effects of explanations on acceptance of deleting proposal, cognitive trust, affective trust, credibility, and uncertainty.

	**Acceptance of deleting proposal^a^**			**Cognitive trust^b^**			**Affective trust^b^**			**Credibility^b^**			**Uncertainty^b^**		
**Predictor**	***Est*.**	***SE***	***z***	***Est*.**	***SE***	***t***	***Est*.**	***SE***	***t***	***Est*.**	***SE***	***t***	***Est*.**	***SE***	***t***
Constant	−0.15	0.36	−0.42	2.24	0.15	14.98^***^	2.30	0.15	15.06^***^	3.09	0.13	23.56^***^	3.83	0.15	24.79^***^
Learning	0.26	0.46	0.55	0.04	0.21	0.85	−0.07	0.21	−0.33	0.04	0.18	0.15	−0.21	0.20	−1.07
Time	0.13	0.03	4.99^***^	0.07	0.01	8.79^***^	0.07	0.01	8.72^***^	0.05	0.01	5.84^***^	−0.09	0.01	−7.93^***^
Familiarity	−0.03	0.18	−0.17	−0.04	0.06	−0.76	0.08	0.06	1.48	0.03	0.06	0.48	−0.19	0.07	−2.50^*^
Explanation	1.13	0.18	6.23^***^	0.23	0.06	4.10^***^	0.27	0.06	4.88^***^	0.43	0.06	7.58^***^	−0.58	0.07	−7.50^***^

*N*_*Level*1_ = 876, *N*_*Level*2_ = 73.

a Logistic multilevel regression analysis.

b Continuous multilevel regression analysis.

*** *p* < 0.001,

* *p* < 0.05.

**Table 9B:** Experiment 3: Effects of explanations on checking the system.

	**Checking the file system^a^**			**Time spent checking the file system^b^^,^ ^c^**		
**Predictor**	***Est*.**	***SE***	***z***	***Est*.**	***SE***	***t***
Constant	2.41	0.77	3.15^**^	6.94	0.55	12.61^***^
Learning	1.24	1.08	1.15	0.80	0.78	1.03
Time	−0.07	0.03	−1.91	−0.11	0.03	−3.91^***^
Familiarity	0.46	0.24	1.92	0.30	0.19	1.63
Explanation	−0.79	0.24	−3.26^**^	−0.54	0.19	−2.92^**^

*N*_*Level*1_ = 876, *N*_*Level*2_ = 73.

a Logistic multilevel regression analysis.

b Continuous multilevel regression analysis.

c Logarithmic (originally in milliseconds).

*** *p* < 0.001,

** *p* < 0.01.

Hypothesis 2 proposed a mediating role of information uncertainty for the relationship between explanations and acceptance of the deletion proposal, trust, and credibility (Hypotheses 2a–c). As expected, we found that explanations reduced information uncertainty, which in turn led to increased acceptance of deletion proposals (indirect effect = 0.07, 95% CI [0.05; 0.09], *p* < 0.001), cognitive trust (indirect effect = 0.23, 95% CI [0.17; 0.30], *p* < 0.001), affective trust (indirect effect = 0.25, 95% CI [0.18; 0.32], *p* < 0.001), and credibility (indirect effect = 0.18, 95% CI [0.12; 0.23], *p* < 0.001). For the frequency of checking the explanations, a further indicator of trust, no indirect effect was found for either opening the file system (indirect effect = 0.00, 95% CI [−0.01; 0.01], *p* = 0.890) or time spent checking the file system (indirect effect = −0.03, 95% CI [−0.13; 0.07], *p* = 0.569).

**Figure 3:**
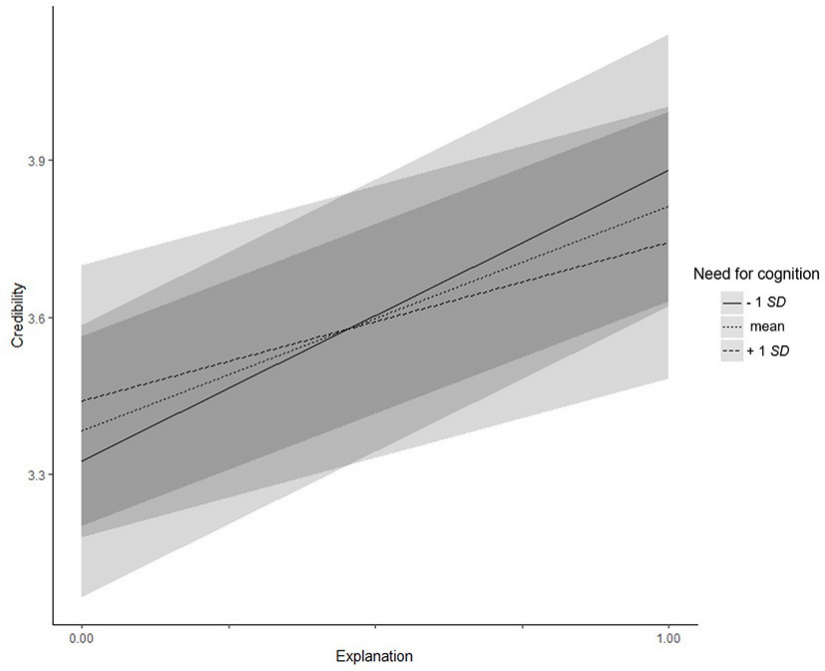
Experiment 3: Interaction effect between need for cognition and explanations on credibility. Low and high levels of need for cognition represent one standard deviation below and above the mean, respectively.

**Figure 4:**
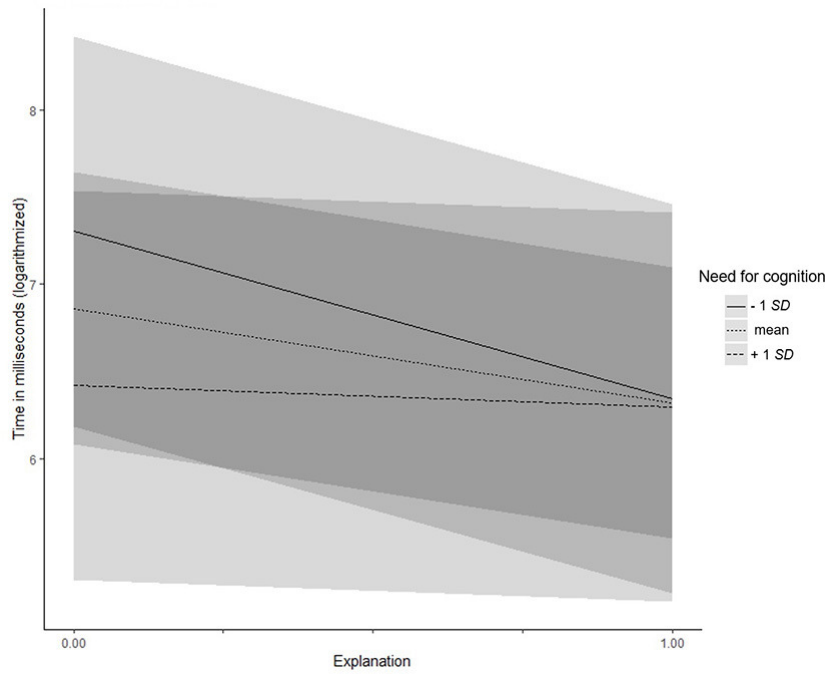
Experiment 3: Interaction effect between need for cognition and explanations on time spent checking the file system. Low and high levels of need for cognition represent one standard deviation below and above the mean, respectively.

Need for cognition (Hypotheses 3a–d) did not moderate the relationship between explanations and acceptance of the proposals, trust, or information uncertainty (all *z*s/*t*s < |1.72|, all *p*s > 0.087), but did moderate the relationship with credibility (γ = −0.31, *SE* = 0.14, *t* = −2.22, *p* = 0.027): Participants with lower (−1 *SD*) need for cognition considered the system as more credible when explanations where given (simple slope = 0.56, *t* = 6.93, *p* < 0.001). For participants with higher (+1 *SD*) need for cognition, the direction of the effect remained the same, but turned out to be weaker (simple slope = 0.30, *t* = 3.79, *p* < 0.001; see Figure 3). Moreover, need for cognition moderated the effect of explanations on the time participants spent checking the file system (γ = 1.05, *SE* = 0.47, *t* = 2.24, *p* = 0.025). Participants with lower (−1 *SD*) need for cognition checked the system for a shorter time when an explanation was provided (simple slope = −0.96, *t* = −3.66, *p* < 0.001), whereas there was no difference for participants with higher (+1 *SD*) need for cognition (simple slope = −0.12, *t* = −0.48, *p* = 0.632, see Figure 4). Hypothesis 3 was partly supported.

For conscientiousness (Hypotheses 4a–d), none of the interaction effects turned out to be significant, all *z*s/*t*s < |1.90|, all *p*s > 0.059. Therefore, Hypothesis 4 was not supported.

Finally, we investigated whether the deletion of files promoted forgetting, particularly for well-known files (learning condition; Hypothesis 5). However, deleting a file was not related to recognition of file names (γ = −0.17, *SE* = 0.19, *z* = −0.90, *p* = 0.368). Therefore, Hypothesis 5 had to be rejected.

##### Additional analyses

As in Experiment 2, explanations led to a higher hit rate for file names (γ = 0.36, *SE* = 0.16, *z* = 2.23, *p* = 0.026). Nor did we find more forgetting (lower hit rate) of file names in the learning condition or a significant interaction between explanations and learning conditions on the hit rates for the file names.

To further explore our data, we analyzed the variables over the course of the experiment. The results revealed that over time, acceptance of the system's deletion proposals generally increased (γ = 0.13, *SE* = 0.03, *z* = 4.99, *p* < 0.001). Moreover, over time, participants trusted the system more both cognitively (γ = 0.07, *SE* = 0.01, *t* = 8.79, *p* < 0.001) and affectively (γ = 0.07, *SE* = 0.01, *t* = 8.72, *p* < 0.001), considered it more credible (γ = 0.05, *SE* = 0.01, *t* = 5.84, *p* < 0.001), felt less information uncertainty (γ = −0.09, *SE* = 0.01, *t* = −7.93, *p* < 0.001), and spent less time checking the file system (γ = −0.11, *SE* = 0.03, *t* = −3.91, *p* < 0.001).

**Figure 5:**
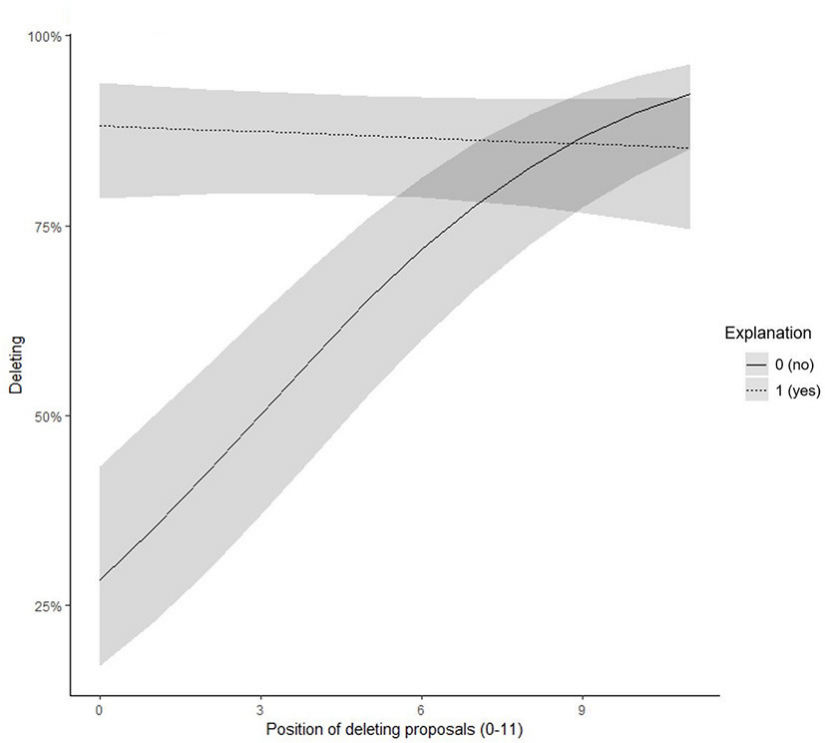
Experiment 3: Interaction effect between position of the deleting proposal and explanations on the probability of accepting the deleting proposal.

We further found a significant interaction effect between time and explanation predicting the probability of accepting the suggestion (γ = −0.33, *SE* = 0.05, *z* = −5.76, *p* < 0.001). When participants received an explanation for the system's suggestion, the probability of acceptance was higher and did not change over time (simple slope = −0.02, *t* = −0.61, *p* = 0.542). However, when the system did not provide an explanation, in the beginning, participants had a low acceptance rate which increased over time (simple slope = 0.31, *t* = 7.12, *p* < 0.001). At the end of the experiment, explanations did not play a role for the acceptance of suggestions (see Figure 5).

In sum, the finding in Experiment 2 that explanations foster acceptance of suggestions, trust, credibility, and information uncertainty could be replicated. Moreover, the mediating role of information uncertainty was confirmed. Again, we found no effect of explanations and deletion of files on participants' memory.

## Discussion

Successfully managing and deleting digital data at work becomes increasingly important. In three experiments, we investigated how individuals respond to an explanatory interactive AI system (Dare2Del), which provides suggestions to delete irrelevant digital objects. To identify important parameters for the user acceptance of these suggestions, we systematically varied several design features within and across the experiments: The presence and kind of explanations as well as the familiarity of the to-be-deleted files were varied in all experiments. In Experiments 2 and 3, we additionally provided the possibility to check the correctness of the provided explanations in the file system. In Experiment 3, we further tested whether it makes a difference if to-be-deleted files are very well known. An overview of the effects of explanations on the outcome variables for all three experiments is provided in Table 10.

**Table 10:** Overview: Hypothesized effects of explanations on dependent variables for Experiments 1, 2, and 3.

	**Experiment 1**	**Experiment 2**	**Experiment 3**
Deleting file^a^	✓	✓	✓
Cognitive trust	x	x	✓
Affective trust	x	x	✓
Checking file^a^^,^ ^b^	–	x	✓
Credibility	x	✓	✓
Uncertainty	x	✓	✓
Recognition^a^	Poorer	Better	Better

a Dichotomous variable.

b Only assessed in Experiments 2 and 3.

Across all experiments, our findings demonstrate a general effectiveness of regularly prompting and supporting users to delete irrelevant data files, as users generally complied with these suggestions in a large number of cases, and deleted the files. Participants deleted even more files when they had the opportunity to check the appropriateness of Dare2Del's suggestions (Experiments 2 and 3). Moreover, our results also highlight the importance of providing explanations: Explanations increased the acceptance of the suggestions in all three experiments. With regard to information uncertainty, trust and credibility, it seems to play an important role whether explanations are comprehensible and can be verified in the system: If this possibility was provided, explanations also decreased information uncertainty (Experiment 2, Experiment 3), resulted in higher trust (Experiment 3) and credibility ratings (Experiment 2, Experiment 3). We assume that the absence of significant trust effects in Experiment 2 was owed to the smaller sample size, which was probably not able to detect the rather small effect.

In all experiments, the familiarity of files had no impact on the acceptance of the suggestions, information uncertainty, trust, and credibility ratings. Moreover, it did not matter whether the digital objects were well-known or not (Experiment 3). One possible explanation is that although the titles were familiar or even well-known, participants did not know details about the content of the documents. The lack of a reference to the content could therefore account for the absence of the hypothesized effects.

We also found that levels of trust, credibility, and information uncertainty changed over time. In Experiment 1, participants surprisingly showed less trust, less credibility, and more information uncertainty over time. We argue that the lack of the system's transparency (i.e., no opportunity to verify deleting suggestions) might have been responsible for these negative effects. However, in Experiment 3 (but not in Experiment 2), we found an increase in trust, credibility, and a decrease in information uncertainty over time. We also found an increase in the acceptance of the system's deleting suggestions without explanations: At the beginning, these suggestions were hardly accepted, but over time, participants gained confidence and increasingly accepted them. We assume that over time, participants were more familiar with the system, felt more confident with its handling and therefore decided to follow its suggestions more often. Whereas explanations helped to overcome uncertainty and build trust in the beginning, they were replaced by experience and inherent trust and therefore no longer needed after a certain period of interaction time.

Information uncertainty mediated effects of explanations on accepting deleting suggestions, trust, and credibility, thus underlining its essential role for the user's acceptance of the system. In contrast, person characteristics such as need for cognition and conscientiousness seem to play a rather minor role—although we found some effects: In Experiment 2, participants with lower conscientiousness reported less information uncertainty when explanations were provided. In Experiment 3, participants with lower need for cognition considered the system as more credible and checked the system for a shorter time when explanations were given. These effects demonstrate that people low in conscientiousness and need for cognition benefit *more* from the presence of explanations: They are more likely to believe the system without questioning or wanting to thoroughly check the adequacy of the deletion suggestions. In contrast, people with high conscientiousness and a high need for cognition may not be convinced by the rather simple explanations we have given, feel more uncertain, and want to check the appropriateness of the explanation on their own (cf. Gajos and Chauncey, 2017; Ghai et al., 2021). Future research might address this issue by systematically varying the level of detail of the provided explanations.

Based on the concept of distributed cognition (Hutchins, 1995), we further investigated whether providing explanations and deleting irrelevant digital objects also promoted the forgetting of related content in human memory. Different patterns of results emerged here: Whereas the presence of explanations led to poorer recognition in Experiment 1, we even found better recognition rates in Experiments 2 and 3. We assume that this is due to the systematic design differences between the experiments: As participants had the opportunity to check the underlying file systems Experiments 2 and 3, they probably studied the file names more intensively. This higher processing depth might have strengthened subsequent recognition and thus counteracted forgetting. Interestingly, accepting deleting suggestions also led to impaired recognition in Experiment 1, but we were not able to replicate these effects in Experiments 2 and 3. The finding suggests that there might be a connection between deleting files from external storage systems and their related internal representations. However, this effect vanishes, when other factors require a deeper processing of the file names to make an informed decision as in Experiments 2 and 3.

### Implications

The findings of our experiments contribute to the existing research both theoretically and practically. First, and in line with prior research (e.g., Pu and Chen, 2007; Mercado et al., 2016), our results confirm that providing explanations is an important and effective design factor, which positively influences the interaction with assistive systems and the acceptance of their suggestions. Within the present research, we also show why explanations can be beneficial to follow the system's suggestions: Explanations can reduce information uncertainty, and therefore lead to more trust and higher acceptance of the presented recommendations. These findings highlight the importance of reducing information uncertainty to enable fast and effective decisions. However, to actually be able to reduce information uncertainty, assistive systems and their suggestions need to be transparent and verifiable (see also Miller, 2018; Muggleton et al., 2018). Our experiments provide empirical evidence to previous considerations and strongly recommend considering comprehensibility and transparency in the future design of interactive AI systems. Thereby, our results suggest that the positive effects of providing explanations for deletion suggestions can even be extended by giving users the possibility to check whether the suggestions are correct. This is a novel feature which is comparably easy to implement, but could have promising effects in terms of user's acceptance and cooperation with assistive systems.

Beyond that, we found no clear evidence of immediate consequences from deleting external computer files on related internal memory representations. Although some of our results (Experiment 1) are in line with the distributed cognition approach (Hutchins, 1995) and suggested a connection between deleting and forgetting, the effect seems to be rather weak and susceptible to many context factors (e.g., memory processing depth, see below). Moreover, other cognitive phenomena such as benefits of cognitive offloading (Risko and Gilbert, 2016) or saving-enhanced memory (Storm and Stone, 2015) could come into play and prevent successful forgetting when actually saved files should be deleted. Further research is needed to elaborate how these phenomena interact, how the organization of our digital work environment and related mental representations are connected, and for whom and when deleting files can ultimately lead to a relief of human memory.

### Strengths, limitations, and further research

A clear strength of the present research lies is the systematic variation of design features within and across experiments. By successively adjusting the system's parameters (i.e., providing the possibility to check suggestions), we were able to identify and elaborate the most important aspects for successful human-computer interactions. Beyond that, we also addressed possible underlying mechanisms and tested possible cross-connections between the external storage of files and related human memory.

Nevertheless, some limitations of the current studies should be noted. First, we only relied on student samples in all experiments. Although using homogeneous samples is not unusual in experimental research to keep possible disruptive factors constant, it limits the variance and the generalizability of the findings: We assume that the examined person variables (need for cognition, conscientiousness) are above average in our sample. This may have prevented the identification of more substantial individual differences.

Second, we tested our hypotheses using one specific context and task across our experiments—namely deleting files. Although this task is widely application-related as most people save (too) many irrelevant digital objects on electronic devices at work and in their private life, the question remains whether our results can be generalized to other tasks and actions. This might be especially valid for the behavioral outcome (i.e., deleting files)—but less for trust and credibility, as these outcomes have already been examined in relation to explanations in different contexts (Pu and Chen, 2007; Pieters, 2011; Shin, 2021). Moreover, in our experiments, participants worked with Dare2Del for about an hour, which may not have been enough time to get familiar with the system or to develop trust and interaction routines. Thus, our results rather reflect interaction processes when new assistive systems are introduced than long-lasting work routines. Future research should investigate the effects of explanations on affective, cognitive and behavioral outcomes for a longer time and with file systems which are much more familiar to the participants.

The third point refers to our measurement of forgetting. It may be that we failed to find a substantial forgetting effect also due to a too short time interval between the main experiment and the recognition task. We already mentioned high processing depth as possible reason for this phenomenon. However, it may be that dealing with a file—which is required to finalize the deleting decision—increases its accessibility in the short term. Nevertheless, it might still help to detach from and forget it in the long term. Therefore, it would be interesting to explore long-term memory effects in future studies. In addition, we used a recognition task. Research on intentional forgetting has shown that forgetting effects are stronger and consistent in free recall tests but not compulsorily in recognition tests (MacLeod, 1975). Thus, future experiments might use also free recall to investigate forgetting.

### Conclusion

The present study examined interactions between humans and interactive computer systems supporting users to delete irrelevant data files. Results underlined the importance of presenting explanations for the acceptance of deleting suggestions, but also point to the need of their transparency and verifiability to generate trust. However, we did not find clear evidence for immediate cross-connections between deleting computer files and human forgetting of the related mental representations.

## Data availability statement

The datasets presented in this study can be found in online repositories. The names of the repository/repositories and accession number(s) can be found below: https://osf.io/dk6en/.

## Ethics statement

The studies involving human participants were reviewed and approved by Deutsche Gesellschaft für Psychologie. The patients/participants provided their written informed consent to participate in this study.

## Author contributions

KG and CN were responsible for study design and wrote the manuscript. KG conducted the data collection and analyzed the data. SS provided technical support for the experimental setup. US contributed to the study design and supported writing the manuscript. All authors contributed to the article and approved the submitted version.

## Funding

The project (NI 1066/4-1) was funded by the German Research Council (Deutsche Forschungsgemeinschaft, DFG).

## Conflict of interest

The authors declare that the research was conducted in the absence of any commercial or financial relationships that could be construed as a potential conflict of interest.

## Publisher's note

All claims expressed in this article are solely those of the authors and do not necessarily represent those of their affiliated organizations, or those of the publisher, the editors and the reviewers. Any product that may be evaluated in this article, or claim that may be made by its manufacturer, is not guaranteed or endorsed by the publisher.

## Supplementary material

The Supplementary material for this article can be found online at: https://www.frontiersin.org/articles/10.3389/frai.2022.919534/full#supplementary-material
