# Supplementary material for: Explanatory machine learning for justified trust in human-AI collaboration: Experiments on file deletion recommendations
Source: Front Artif Intell. 2022 Nov 23;5:919534. doi: 10.3389/frai.2022.919534 (PMC9727201; doi:10.3389/frai.2022.919534)
Supplement: Supplementary file 1 [file Data_Sheet_1.PDF]

Supplementary Material 1

Screenshot of the literature management system

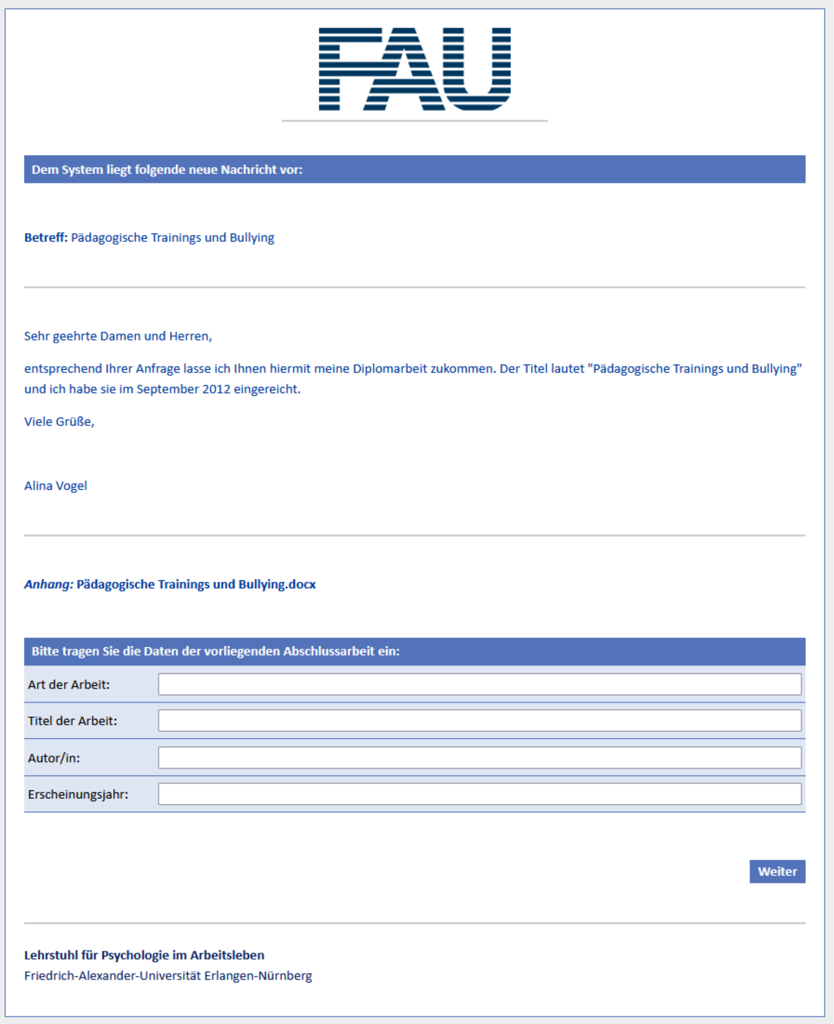

FAU

Dem System liegt folgende neue Nachricht vor:

Betreff: Pädagogische Trainings und Bullying

Sehr geehrte Damen und Herren,

entsprechend Ihrer Anfrage lasse ich Ihnen hiermit meine Diplomarbeit zukommen. Der Titel lautet "Pädagogische Trainings und Bullying" und ich habe sie im September 2012 eingereicht.

Viele Grüße,

Alina Vogel

Anhang: **Pädagogische Trainings und Bullying.docx**

Bitte tragen Sie die Daten der vorliegenden Abschlussarbeit ein:

Art der Arbeit:	
Titel der Arbeit:	
Autor/in:	
Erscheinungsjahr:	

Weiter

Lehrstuhl für Psychologie im Arbeitsleben
Friedrich-Alexander-Universität Erlangen-Nürnberg

Supplementary Material 2

Screenshot of a deleting suggestion of the assistive system Dare2Del

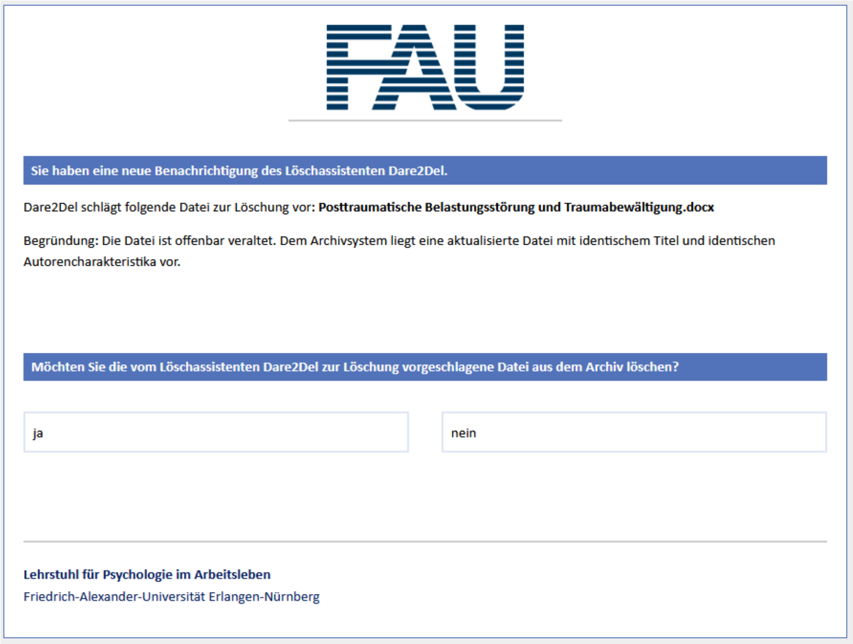

Supplementary Material 3

Screenshot of checking the file system

Lokaler Speicher / Dokumentenarchiv / Abschlussarbeiten

Name	*Änderungsdatum*	*Typ*	*Größe*
Agitation als medizinischer Notfall.docx	04.02.2021	Word	45 KB
Alexithymie in Partnerschaften.docx	07.05.2019	Word	82 KB
Anankastische Persönlichkeitsstörung .docx	03.04.2020	Word	31 KB
Antero- und retrograde Amnesien.docx	05.09.2020	Word	43 KB
Antipsychotika und sexuelle Apetenzprobleme.docx	03.03.2021	Word	64 KB
Aufmerksamkeitsdefizitstörung und Borderline .docx	19.10.2020	Word	59 KB
Benzodiazeptine im Vergleich.docx	20.01.2021	Word	28 KB
Big 5 Persönlichkeitseigenschaften in Deutschland.docx	08.12.2020	Word	39 KB
Desorientiertheit als Corona-Spätfolge.docx	09.02.2021	Word	46 KB
Die Opponent-Process-Theory und Stressmarker.docx	19.02.2020	Word	55 KB
Dopaminrezeptoren und Leistungssport.docx	04.11.2019	Word	67 KB
Endogene Psychosen und Genesungswege.docx	24.08.2019	Word	96 KB
Erlernte Hilflosigkeit als Wirkverstärker bei Depressionen.docx	29.06.2020	Word	42 KB
Generalisierte Angststörung und Entspannungsverfahren.docx	13.04.2022	Word	32 KB
Halluzinationen und Alltagsbewältigung.docx	19.09.2020	Word	23 KB
Herzfrequenzvariabilität als Corona-Warnsignal.docx	09.02.2021	Word	45 KB
Impulsive Handlungen von Pflegebedürftigen.docx	03.07.2019	Word	37 KB
Impulskontrolle und Immunreaktionen.docx	13.04.2022	Word	37 KB
Inzidenzraten und der R-Wert.docx	03.12.2020	Word	20 KB
Klassifikationsprobleme im ICD-10.docx	31.07.2019	Word	43 KB
Kognitionspsychologie und bildgebende Verfahren .docx	30.12.2020	Word	61 KB
Kommunikation von Coronamaßnahmen.docx	27.04.2020	Word	52 KB
Längsschnitt- und Mehrebenenanalysen im Vergleich.docx	23.01.2021	Word	32 KB
Leistungsmotiv und Attribution.docx	13.04.2022	Word	57 KB
Motorische Einschränkungen bei Kleinkindern.docx	01.06.2020	Word	58 KB
Muskelrelaxation bei Angstpatienten.docx	13.11.2019	Word	38 KB
Neurotransmitter und ihre Antagonisten.docx	18.02.2021	Word	49 KB
Obstruktive Schlafapnoe und körperliche Belastbarkeit.docx	09.03.2021	Word	33 KB
Organisationale Veränderungen zu Pandemiezeiten.docx	05.06.2020	Word	42 KB
Organisationsstrukturen und Homeoffice.docx	14.10.2019	Word	43 KB
Paranoia als gesellschaftliches Phänomen.docx	17.07.2020	Word	64 KB
Pausengestaltung im Homeoffice.docx	15.06.2020	Word	67 KB
Plus- und Minussymptome bei Schizophrenie.docx	08.07.2019	Word	37 KB
Posttraumatische Belastungsstörung und Traumabewältigung.docx	04.01.2021	Word	84 KB
Posttraumatische Belastungsstörung und Traumabewältigung(1).docx	13.04.2022	Word	86 KB
Privatsphäre im Großraumbüro.docx	01.02.2021	Word	49 KB
Probleme von Oversampling.docx	22.03.2020	Word	42 KB
Psychoonkologie und Familientherapie.docx	13.04.2022	Word	29 KB
Rauchentwöhnung und Reizkonfrontation.docx	13.04.2022	Word	56 KB
Rehabilitation von Rheumapatienten.docx	30.01.2021	Word	58 KB
Resilienz und Berufserfolg.docx	13.04.2022	Word	33 KB
Schizoaffektive Störungen im Vergleich.docx	22.08.2020	Word	34 KB
Sedativa im Straßenverkehr.docx	15.04.2020	Word	50 KB
Selbstenthüllung und Sympathie.docx	29.07.2020	Word	93 KB
Selbstkonzept und Berufswahl.docx	13.04.2022	Word	73 KB
Soziale Isolation zu Pandemiezeiten.docx	30.03.2019	Word	43 KB
Soziale Unterstützung und Stresserleben.docx	13.04.2022	Word	59 KB
Trennungsangst und selbstverletzendes Verhalten.docx	13.04.2022	Word	42 KB
Überwachung am Arbeitsplatz.docx	07.02.2021	Word	82 KB
Vaskuläre Demenz und Alzheimer.docx	01.10.2020	Word	48 KB

Schließen
